# Case report: fatal pneumonia caused by new sequence type *Legionella* pneumophilia serogroup 1

**DOI:** 10.1097/MD.0000000000022812

**Published:** 2020-10-23

**Authors:** Luxi Jiang, Sixu Tao, Deguang Mu, Naxin Zhang, Li Zhao, Yu Chen

**Affiliations:** a Department of Pulmonary and Critical Care Medicine, Shengjing Hospital of China Medical University, Shenyang, Liaoning; b Department of Pulmonary Critical Care Medicine, Zhejiang Provincial People's Hospital, People's Hospital of Hangzhou Medical College, Hangzhou, Zhejiang; c Department of Pulmonary Critical Care Medicine, Tianjin Third Central Hospital, Tianjin, China.

**Keywords:** case report, legionella, Legionnaires’ disease, pneumonia

## Abstract

**Introduction:**

Legionnaires’ disease is caused by Legionella bacteria, and commonly manifests as pneumonia and has a high fatality rate.

**Patient concerns:**

This case study reports on the fatal incident of a patient, initially diagnosed with pneumonia, and subsequently diagnosed with Legionnaires’ disease caused by a new sequence type (ST) of Legionella.

**Diagnosis:**

It is speculated that the patient acquired Legionnaires’ disease from a contaminated water source. Legionnaires’ disease was diagnosed using the Legionella urinary antigen assay and bacterial cultures of respiratory secretions; Legionella pneumophilia Type 1 was also identified through serological testing. Sequence-based typing of the cultured bacterium revealed it to be a previously unidentified species, and it was named ST2345 new-type.

**Interventions:**

In addition to the treatment of Legionnaires’ disease, blood samples taken on the second day of admission showed a co-infection of Candida tropicalis, which was treated with anti-fungal treatment. The patient improved after a week, however, on the seventh day of administration lower respiratory secretions showed the growth of Klebsiella pneumonia, indicative of ventilator-associated pneumonia.

**Outcomes:**

Despite active treatment, the patient passed away due to multiple organ failure. As this was a fatal case, further research is needed to determine whether the critical condition of this case was related to the virulence of the novel Legionella strain.

**Conclusion:**

A key finding of this study is that treatment for suspected Legionnaires’ disease must be administered rapidly, as infection with Legionella may give rise to secondary pathogenic infections.

## 1 Introduction

Legionnaires’ disease is a disease caused by *Legionella* bacteria that primarily presents as pneumonia and can affect multiple organs. It commonly manifests as severe pneumonia and has a high case fatality rate. Due to its complex and diverse clinical presentation, imaging and routine laboratory tests lack the required specificity, and hence Legionnaires’ disease is prone to misdiagnosis and improper treatment.^[1]^ Here, we report a case of a new sequence type (ST) of fatal Legionnaires’ disease diagnosed using the *Legionella* urinary antigen assay and bacterial culture of respiratory secretions. As this was a fatal case, this paper aims to investigate the cause of death of this patient with Legionnaires’ disease, summarize the experience of treatment failure, and report on this new-ST *Legionella*.

## 2 Case presentation

### 2.1 Chief complaints

The patient was a 59-year-old male who was admitted to our hospital on July 19, 2012 due to “fever with progressive dyspnea for 6 days.”

### 2.2 History of present illness

The patient developed a high fever 6 days prior and his body temperature reached a maximum of 39.6°C. He also presented with a cough, yellow sputum, and bloody sputum, accompanied by progressive dyspnea. His body temperature dropped after intravenous infusion of cefoxitin and dexamethasone but rose again, and he was admitted to our hospital for treatment.

### 2.3 History of past illness

Two weeks before the onset of the disease, the patient received root canal treatment (including drilling and rinsing) at a private dental clinic due to toothache. He had a history of cirrhosis, had been smoking for 30 years, and his smoking index was 30 pack-years.

### 2.4 Physical examination upon admission

T36.9°C, P140 beats/minute, R22 beats/minute, BP119/80 mmHg, SO_2_ 78%, clear consciousness, cyanotic lips, extensive moist rales in both lungs, heart rate 140 beats/minute, regular rhythm, soft abdomen, no tenderness, and edema of both lower extremities.

### 2.5 Laboratory examinations

Blood routine: white blood cells 3.1 × 10^9^/L, neutrophil percentage 77%, hemoglobin 110 g/L, platelets 10^8^ × 10^9^/L; pro-brain natriuretic peptide 1271 pg/mL, creatine kinase 94 U/L, troponin 0.34 ng/mL; albumin 22.1 g/L, alanine aminotransferase 153 U/L, aspartate aminotransferase 580 U/L, total bilirubin 47.4 mmol/L; creatinine 121.5 mmol/L, urea 14.6 mmol/L; blood sodium 126 mmol/L; blood gas analysis (aerobic state): pH 7.32, PCO_2_ 49 mmHg, PO_2_ 33 mmHg, lactic acid 3.2mmol/L; positive *Legionella* urinary antigen assay.

### 2.6 Imaging examinations

**Figure 1:**
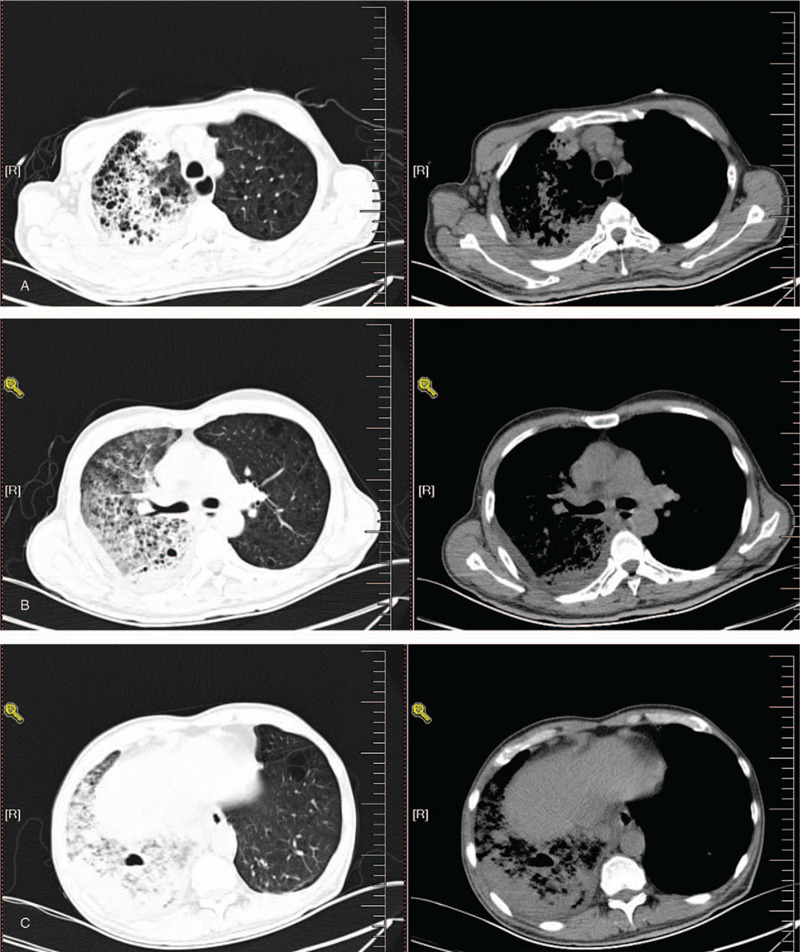
A–C, Pulmonary CT upon admission. Diffuse ground-glass opacity of the right lung with lower lobe consolidation; right lower lobe cavities with fluid level; right pleural effusion.

A pulmonary computed tomography (CT) scan showed diffuse ground-glass opacity of the right lung with lower lobe consolidation; right lower lobe cavities with fluid level; right pleural effusion (Fig. 1).

### 2.7 Microbiological identification of the causative agent

**Figure 2:**
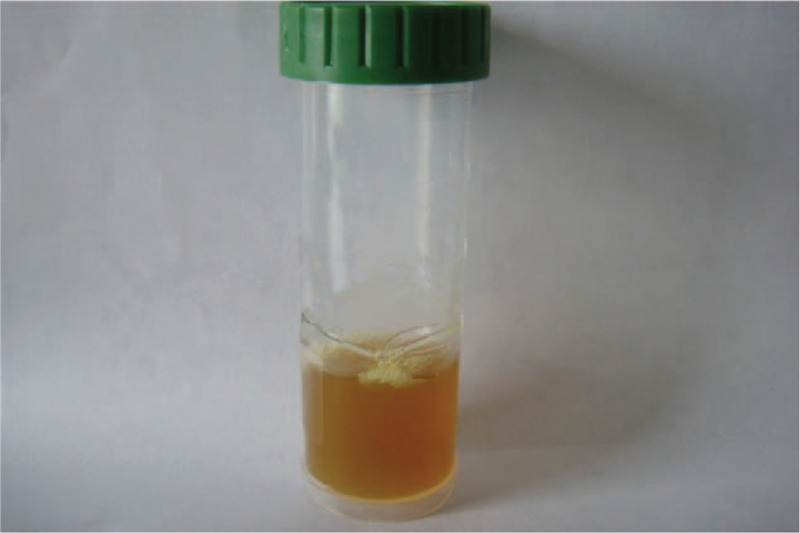
Lower respiratory tract secretion. The lower respiratory tract secretion aspirated through endotracheal intubation was orange-yellow in color.

**Figure 3:**
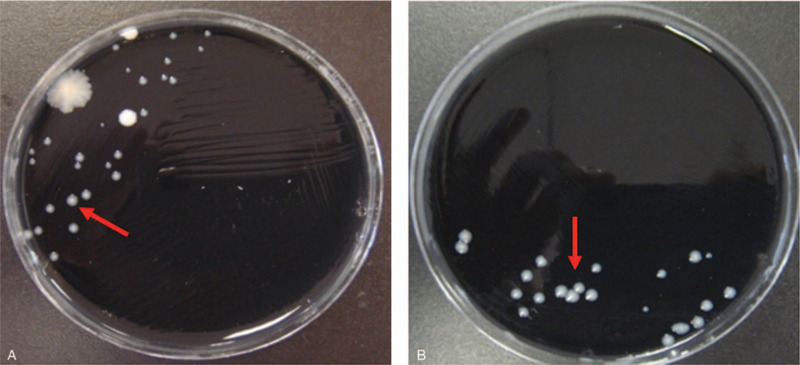
A-B, *Legionella* culture. Growth of *Legionella* colonies can be seen on buffered charcoal yeast extract (BCYE)(3A) and *Legionella*-selective Glycine Vancomycin Polymyxin Cycloheximide (GVPC) (3B) media (red arrows indicate typical *Legionella*).

**Figure 4:**
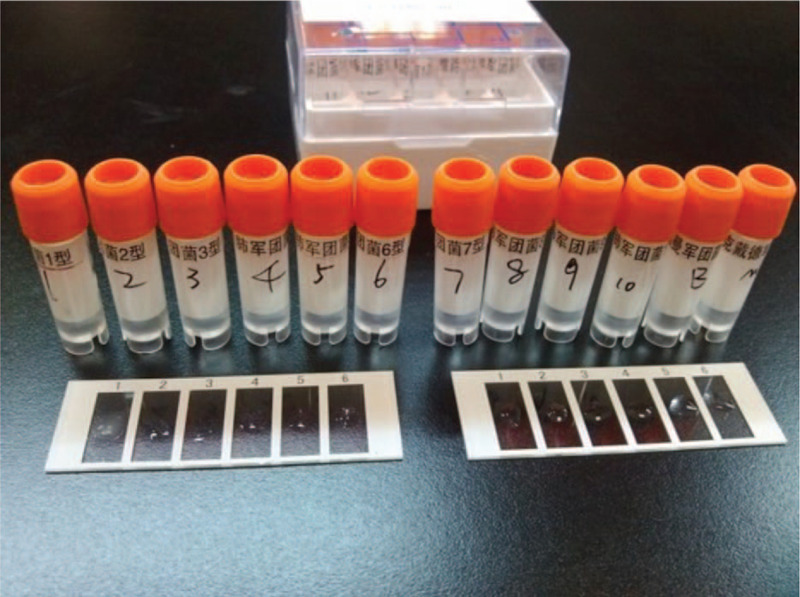
*Legionella* serological identification (slide agglutination method). Serological identification of the cultured *Legionella* was performed using the slide agglutination method. The leftmost slide (No. 1) shows that agglutination reactions occurred between this *Legionella* strain and *Legionella pneumophila* Type I, and the mixture shows sand-like changes. This *Legionella* strain did not agglutinate with other serotypes, and the mixture remained clear.

After admission, the lower respiratory tract secretion aspirated through endotracheal intubation was orange-yellow in color (Fig. 2). The secretion was positive for *Legionella* cultured in buffered charcoal yeast extract (BCYE) agar and *Legionella*-selective Glycine Vancomycin Polymyxin Cycloheximide (GVPC) agar (Fig. 3). Serological testing identified the strain as *Legionella pneumophila* Type I (Fig. 4). The blood samples on the second day of admission showed the growth of *Candida tropicalis*, and serum *Legionella* antibody IgM was positive on the ninth day of admission. *L pneumophila* Type I was cultured and isolated from the respiratory secretions of this patient, which were sent for further sequence-based typing (SBT). More specifically, PCR amplification was performed on this *Legionella* strain, and seven *L pneumophila* housekeeping gene loci were detected: fl*aA*, *pilE*, *asd*, *mip*, *mompS*, *proA*, and *neuA*. This sequencing result was compared to the SBT database of the European Working Group for Legionella Infections (http://www.ewgli.org), and the specific housekeeping allelic profile was: 2, 10, 57, 28, 2, 1, and 6. The results indicate that this *Legionella* strain is a new sequence type (ST) that has not been reported internationally. The sequencing result of this strain was uploaded to the database, and the strain was named ST2345 new-type.

#### 2.7.1 Diagnosis

After admission, the patient was diagnosed with severe community-acquired pneumonia.

#### 2.7.2 Treatment

**Figure 5:**
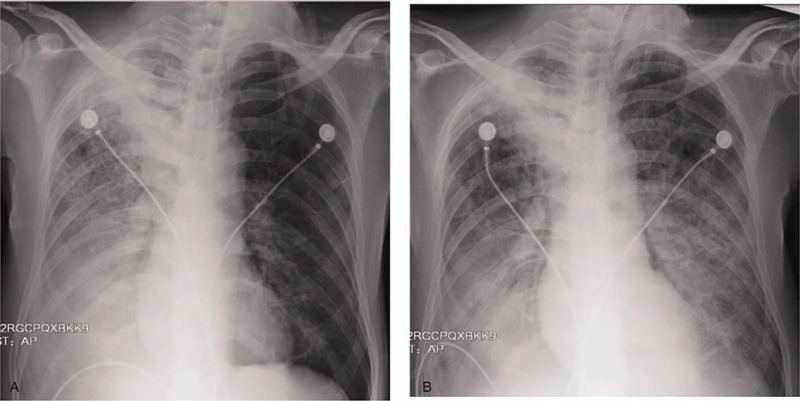
A-B, Chest X-ray upon admission (July 21) (5A) and after admission (July 26) (5B). Absorption of right lung primary lesions; exudative lesions in the left lung.

As oxygenation could not be maintained by oxygen mask and non-invasive ventilation, mechanical ventilation was performed via endotracheal intubation. Imipenem/cilastatin was combined with moxifloxacin intravenous infusion for anti-infective treatment. The patient developed septic shock after admission and was given symptomatic vasopressors and organ support therapy. The patient was positive for *Legionella* urinary antigen, which suggested the patient had contracted Legionnaires’ disease, and doxycycline nasal combination therapy was administered. The patient's blood culture suggested the growth of *Candida tropicalis* (*C tropicalis)*, and voriconazole antifungal treatment was added. After 1 week of treatment the patient's condition had improved slightly, the septic shock was corrected, and the vasopressor was gradually reduced and stopped. However, the patient's body temperature did not return to normal, fluctuating between 37°C and 38.5°C. Furthermore, his oxygenation was poor, and the concentration of inhaled oxygen had to be above 70% to ensure that his PO2 remained at approximately 60 mmHg; the lower respiratory tract secretions gradually turned from orange to bloody. The patient developed high fever again on the seventh day of admission and his body temperature was 40°C. Culture of the lower respiratory secretions showed the growth of *Klebsiella pneumoniae*, elevated procalcitonin 54.77 ng/mL, and endotoxin 201.4 pg/mL. Repeat bedside chest X-ray revealed the absorption of the primary lesions in the right lung, and exudative lesions in the left lung (Fig. 5). Secondary ventilator-associated pneumonia was considered. Despite active treatment, the patient passed away due to multiple organ failure.

## 3 Discussion

Legionnaires’ disease often develops into severe pneumonia. Nearly 50% of hospitalized patients with *Legionella* infections need to be admitted to the ICU, and its case fatality rate is between 5% to 30%.^[2]^ The patient in this case presented with multiple organ failure upon admission, and subsequently developed septic shock. Although anti-Legionella drugs (moxifloxacin, doxycycline) were administered, the patient continued to have a high fever, which still could not be brought down to normal levels using an ice blanket. Causes of the high fever may include: 1. Uncontrolled *Legionella* infection. Despite the use of moxifloxacin and oral doxycycline, international guidelines indicate that patients with severe *Legionella* infection may have a poor prognosis for monotherapy and should receive combination therapy,^[3]^ whereas oral doxycycline has a weaker efficacy, which may be less effective than combined intravenous medication. 2. Secondary double infections may have caused long-term fever. On the second day of admission (40 hours after admission), the patient's blood culture showed a positive result that indicated the growth of *C tropicalis*. In the later stages of treatment (seventh day of hospital stay), the culture of lower respiratory tract secretions indicated the growth of *K pneumoniae*, presence of C-reactive protein, an elevated procalcitonin index compared to before, and appearance of ventilator-associated pneumonia. This patient contracted both fungal and bacterial secondary infections, which led to his poor prognosis. There have been many reports on secondary infections in Legionnaires’ disease. As early as 1979, Gump et al. reported the emergence of infections by various pathogens in patients with Legionnaires’ disease, including *K pneumoniae*, *Staphylococcus aureus*, and *Enterobacter*.^[4]^ In the same year, Cohen et al. found among 10 cases of fatal nosocomial Legionnaires’ disease that 4 cases had positive sputum cultures; the positive bacteria were *S aureus*, *K pneumoniae*, and *Citrobacter*.^[5]^ In 2002, Tan et al reported 6 cases of Legionnaires’ disease with other bacteremic coinfections and inferred that patients with Legionnaires’ disease are prone to bacteremia.^[6]^ Our research group found in animal experiments of Legionnaires’ disease that other non-*Legionella* bacteria could be cultured from the lungs and liver of mice during the acute phase of the disease, which mainly included *Escherichia coli*, *S aureus*, and *Citrobacter*, whereas the control group showed no bacterial growth. Hence, our findings further validate this view.^[7]^ Currently, the mechanisms underlying how Legionnaires’ disease gives rise to other secondary pathogenic infections is unknown. There is a hypothesis that the translocation of intestinal bacteria may occur during the acute phase of Legionnaires’ disease.^[5]^ Therefore, the issue of double infections in patients with *Legionella* infection should not be ignored, and close attention should be paid to double infections, early discovery, and early active treatment. With respect to the high fever caused by double infections, when the patient's condition deteriorates, we should apply hemofiltration in the early stages in addition to antibiotics. This is because there is an outbreak of inflammatory mediators during the acute phase of *Legionella* infection, which will cause elevated levels of inflammatory mediators, thus resulting in patients with persistently high fever and poor condition.^[8–11]^ Hemofiltration can remove inflammatory mediators and might improve prognosis.

The patient in this case had a root canal treatment two weeks before disease onset and had a history of dental restoration. The source of infection may have been water contaminated by *Legionella* in the pipelines of the dental clinic. We isolated and cultured the pipe water from the pipeline network in the dental clinic, but the results were negative. As there was a delay between the time of our isolation and culture and the patient's disease onset, we believe that the dental clinic may have disinfected the water source, which led to our negative results. This case suggests that we should trace the history of suspected environmental exposure in cases of suspected Legionnaires’ disease, such as air-conditioned environments, hot spring bathing, pipeline repairs, exposure to contaminated drinking water, rainwater, etc.^[12,13]^ This case also suggests that dental clinics and similar venues should regularly disinfect their water source to prevent *Legionella* infections in patients.

Due to the rapid development of Legionnaires’ disease, inappropriate initial treatments can significantly increase the patient's fatality rate. The cause of death in this patient was related to his delayed diagnosis and delayed treatment. As the treatment for *Legionella* was not administered before the patient was hospitalized, this meant he had missed the window for early treatment and his condition progressed rapidly, manifesting as severe pneumonia with severe respiratory failure and multiple organ involvement. Thus, the subsequent treatment for *Legionella*, which was administered after the patient was hospitalized, was not effective. Therefore, we urge physicians to pay closer attention to the clinical presentation of such patients and to the features of suspected *Legionella* cases. If cases of adult community-acquired pneumonia show clinical symptoms, such as fever with bradycardia, acute onset headache, non-drug-induced disturbance of consciousness/lethargy, non-drug-induced diarrhea, shock, acute liver and kidney function damage, hyponatremia, hypophosphatemia, and no response to β-lactam antibiotics, then the possibility of Legionnaires’ disease should be considered.^[14–21]^ This patient was diagnosed with severe pneumonia with rapid disease progression, manifesting as severe respiratory failure with multiple organ involvement and hyponatremia. Thus, *Legionella* infection should be suspected. Furthermore, we found that the patient's sputum was a distinct orange-yellow color (Fig. 2). We have also previously observed that the sputum of patients with Legionnaires’ disease similarly presented this distinct color. Therefore, such orange-yellow sputum may be instructive in the clinical diagnosis of *Legionella* infection.

This patient was suspected of *Legionella* infection after admission. Thus, *Legionella* urinary antigen assay and respiratory secretion culture were performed immediately, which confirmed the pathogenic diagnosis as a *L pneumophila* Type 1 infection. The *L pneumophila* Type I isolated from this respiratory secretion culture was further subjected to sequence-based typing (SBT). The sequencing results were recorded on the SBT database of the European Working Group for Legionella Infections (http://www.ewgli.org), and the strain was named ST2345 new-type. The SBT method is a gene sequence typing technique for *Legionella* that is currently widely applied in the molecular typing of *Legionella*. Most of the *Legionella* cases reported internationally are of the ST1 type. The *Legionella* strain cultured from this patient was named ST2345 new-type, which implies that this is the first discovery of this ST internationally. It also indicates that the *Legionella* contracted by this patient has unique features. As this is a fatal case, further research is needed to verify whether the critical condition of this case was related to the high virulence of this strain.

## Author contributions

**Conceptualization:** Luxi Jiang.

**Data curation:** Deguang Mu, Naxin Zhang.

**Formal analysis:** Deguang Mu, Naxin Zhang, Li Zhao.

**Funding acquisition:** Yu Chen.

**Methodology:** Yu Chen, Deguang Mu, Li Zhao.

**Writing – original draft:** Luxi Jiang, Sixu Tao.

**Writing – review & editing:** Sixu Tao, Yu Chen.
